# Overexpression of m^7^G writers METTL1 and BUD23 confers oncogenicity in kidney renal clear cell carcinoma

**DOI:** 10.1002/path.6453

**Published:** 2025-07-18

**Authors:** Anni Su, Jessica Tieng, Xueying S Xu, Richard P Tan, Yuchen Feng, Justin J‐L Wong

**Affiliations:** ^1^ Epigenetics and RNA Biology Laboratory, Charles Perkins Centre University of Sydney Camperdown Australia; ^2^ School of Medical Sciences, Faculty of Medicine and Health University of Sydney Camperdown Australia

**Keywords:** kidney cancer, RNA modification, m^7^G methylation, METTL1, BUD23, oncogene, tumor suppressor gene

## Abstract

Emerging evidence shows that N7‐methylguanosine (m^7^G) modification and its writers contribute to the development of diverse cancers. However, the role of m^7^G writers in kidney renal clear cell carcinoma (KIRC) remains unclear. In this study we show that the catalytic components of m^7^G writers, METTL1 and BUD23, are overexpressed in advanced KIRC and are associated with worse overall survival. Knockdown of *METTL1* or *BUD23* inhibited cell proliferation, colony formation, and migration in KIRC cell lines, indicating their oncogenic role in KIRC. Furthermore, we observed that *METTL1* and *BUD23* expression was negatively correlated with the expression of key tumor suppressor genes commonly dysregulated in KIRC. We observed METTL1‐mediated m^7^G methylation in mRNAs expressed by these tumor suppressor genes, indicating that METTL1 may regulate these genes *via* m^7^G modification. Overall, our study highlights the oncogenic role of METTL1 and BUD23 in KIRC and their potential as prognostic biomarkers and therapeutic targets in KIRC. © 2025 The Author(s). *The Journal of Pathology* published by John Wiley & Sons Ltd on behalf of The Pathological Society of Great Britain and Ireland.

## Introduction

Kidney renal clear cell carcinoma (KIRC) is the dominant subtype that constitutes up to 80% of all kidney cancers [1]. Current treatments for advanced KIRC, including combination therapy with tyrosine kinase inhibitors (TKIs), targeting the VEGF signaling and immune checkpoint inhibitors (ICIs), have shown efficacy but are limited by drug toxicities and high recurrence rates [1, 2, 3]. Identifying new therapeutic targets is essential to improve outcomes in advanced KIRC.

N7‐methylguanosine (m^7^G) is one of the most prevalent RNA modifications in eukaryotes. It is essential for gene expression control by regulating RNA processing, stabilization, maturation, and translation [4, 5, 6, 7, 8, 9]. In humans, internal m^7^G methylation in messenger RNA (mRNA), transfer RNA, and microRNA are methylated by the methyltransferase 1, tRNA methylguanosine (METTL1)/tRNA N7‐guanosine methyltransferase noncatalytic subunit (WDR4) complex, where METTL1 and WDR4 serve as the catalytic and the regulatory subunit respectively [4, 6]. Another m^7^G methyltransferase complex, BUD23 rRNA methyltransferase and ribosome maturation factor (BUD23), formerly known as WBSCR22/tRNA methyltransferase activator subunit 11–2 (TRMT112) methyltransferase, deposits m^7^G at G1639 in 18S rRNA, with BUD23 functioning as a catalytic subunit and TRMT112 maintaining its metabolic stability [8]. Recent studies have highlighted the pathological role of overexpressed METTL1 and BUD23 in the progression in several cancer types including brain cancer, liver, and prostate cancers [10, 11, 12, 13]. However, their roles in KIRC remain to be determined.

Analysis of data from The Cancer Genome Atlas Research Network (TCGA) suggests that dysregulation of m^7^G writers is associated with overall survival (OS) in KIRC [14]. Whether aberrant expression of m^7^G writers drives KIRC growth and metastatic potential has not been experimentally validated. Here, we further demonstrate that overexpression of *METTL1* and *BUD23* is associated with worse OS in late‐stage KIRC patients. Knockdown of *METTL1* and *BUD23* in KIRC cell lines significantly impairs cancer cell growth, colony‐forming capacity, and migration *in vitro*, indicating that overexpression of these genes encoding m^7^G writers confers oncogenic potential in KIRC. 3D culture models demonstrate the impairment of tumor mass formation following *METTL1* and *BUD23* knockdown. Furthermore, our bioinformatic analysis provides insights into the association between *METTL1* and aberrant expression of tumor suppressor genes (TSGs) that may be regulated *via* m^7^G in KIRC.

## Materials and methods

### Computational analysis

The RNA‐seq dataset for KIRC primary tumors (*n* = 541) and associated clinical information were obtained from TCGA (https://portal.gdc.cancer.gov/) *via* the R package ‘TCGAbiolinks’ [15]. Gene expression analysis was stratified by the clinical stage in KIRC, and statistical significance was determined using analysis of variance (ANOVA) (*p* < 0.05).

OS analysis was performed by comparing KIRC tumors with expression of *METTL1* and *BUD23* above (high) and below (low) the median levels. The log‐rank test and Kaplan–Meier curves were generated using ‘survival’ (https://CRAN.R-project.org/package=survival) and ‘survminer’ (https://rpkgs.datanovia.com/survminer/index.html) R packages. Univariate and multivariate Cox proportional hazards regression models were employed, with *p* value <0.05 considered statistically significant.

The correlation of expression levels between *METTL1*, *BUD23*, *PBRM1*, *PTEN*, and *SETD2* in KIRC tumors was determined using the Pearson correlation coefficient.

m^7^G methylated RNA immunoprecipitation sequencing (m^7^G‐MeRIP‐seq) data were obtained from GSE112276 [9]. Details on m^7^G‐MeRIP‐seq analysis are described in the Supplementary materials and methods.

### Cell culture

CAKI‐1 cells and 786‐O cells were cultured in RPMI 1640 Medium supplemented with HEPES (ThermoFisher, Waltham, MA, USA),10% FBS (Bovogen, East Keilor, Victoria, Australia), 1 mm sodium pyruvate (ThermoFisher), and 1% penicillin/streptomycin (ThermoFisher).

Details on knockdown vector cloning, lentivirus production, and transduction are described in the Supplementary materials and methods. shRNA sequences are listed in supplementary material, Table S1. Transduced cells expressing the knockdown vector containing the puromycin resistance gene were selected in media with puromycin (ThermoFisher) (2 μg/ml for 786‐O and 1 μg/ml for CAKI‐1) for at least 7 days. After puromycin selection, the CCK8, colony formation, wound healing, and 3D tumor mass formation assays were performed (see Supplementary materials and methods).

### Reverse‐transcription quantitative PCR (RT‐qPCR)

Total RNA was extracted from CAKI‐1 and 786‐O cells using the TRIzol™ Reagent (Invitrogen, La Jolla, CA, USA). The cDNA was synthesized with iScript™ gDNA Clear cDNA Synthesis Kit (Bio‐Rad, Hercules, CA, USA). RT‐qPCR was performed using SensiFAST SYBR No‐ROX kit (Bioline, Memphis, TN, USA) with cycling conditions of 95 °C (3 min), 95 °C (10 s), 60 °C (30 s), and 72 °C (20 s) for 40 cycles. Primer sequences are detailed in the supplementary material, Table S2.

### Western blotting

For western blotting, proteins lysates were prepared with RIPA buffer (20 mm Tris–HCL, 150 mm NaCl, 1 mm EDTA, 1 mm EGTA, 1% (w/v) Triton X‐100, pH 7.5), 1% protease inhibitor (ThermoFisher), and 2% phosphatase inhibitor (Cell Signaling Technology, Danvers, MA, USA). Protein concentrations were determined using Micro BCA™ Protein Assay Kit (Life Technologies, Carlsbad, CA, USA). Twenty μg of samples were loaded onto the NuPAGE™ 4%–12%, Bis‐Tris gel (Life Technologies) and transfer to PVDF membranes (Millipore, Burlington, MA, USA). Membranes were blocked in 5% milk and incubated with primary and secondary antibodies (supplementary material, Table S3) before visualizing using SuperSignal West Pico PLUS Chemiluminescent Substrate (ThermoFisher) on a ChemiDoc™ Imaging System (Bio‐Rad).

## Results and discussion

**Figure 1:**
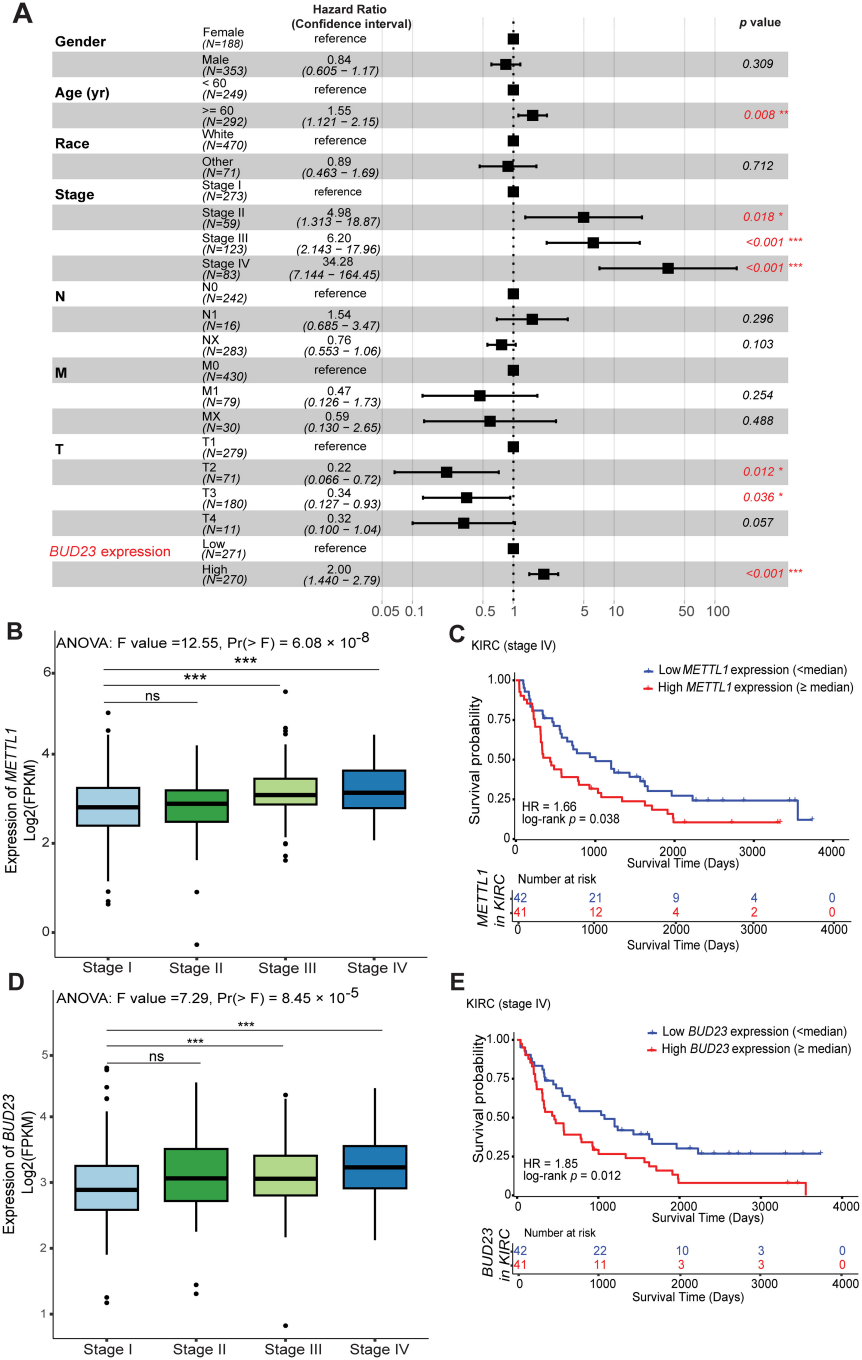
Gene expression and clinical relevance of *METTL1* and *BUD23* in KIRC. (A) Multivariate analysis of *BUD23* against other key clinicopathological features in KIRC. Significance is denoted by *p* < 0.05 with log‐rank text. (B) Expression levels of the *METTL1* in KIRC across different clinical stages. Significance is denoted by *p* < 0.05 by ANOVA test followed by Dunnett's test for multiple comparison of the means. (C) Kaplan–Meier survival curves of KIRC patients based on the expression of *METTL1*. Red and blue represent higher (> median) and lower (< median) levels of gene expression, respectively. Significance is denoted by *p* < 0.05 by log‐rank test. (D, E) Gene expression and survival analysis (similar to panels (B) and (C)) were performed for *BUD23* using data from the TCGA KIRC cohort.

Our previous univariate analysis performed on the TCGA KIRC cohort demonstrated that *METTL1* and *BUD23* are upregulated in primary KIRC compared to normal kidney tissues [14]. Higher expression levels of these genes are associated with worse OS, indicating their potential as prognostic biomarkers in KIRC [14]. To evaluate whether METTL1 and BUD23 serve as independent prognostic factors, we conducted multivariate Cox regression analysis against clinicopathological features [gender, age, race, clinical stage, Tumor (T), Node (N), and Metastasis (M)] using the TCGA KIRC cohort. This analysis showed that upregulation of *BUD23* is an independent prognostic factor in KIRC (Figure 1A). Although *METTL1* upregulation did not appear to be a significant prognostic predictor against other clinical parameters in the KIRC cohort (supplementary material, Figure S1), it is significantly associated with a worse OS in advanced clinical stage (Stage IV) of KIRC (Figure 1B,C). Similarly, *BUD23* is upregulated in late‐stage KIRC, further linking it to poor prognosis (Figure 1D,E). Overall, these findings indicate that upregulated *METTL1* and *BUD23* may contribute to worse clinical outcome in late‐stage KIRC.

**Figure 2:**
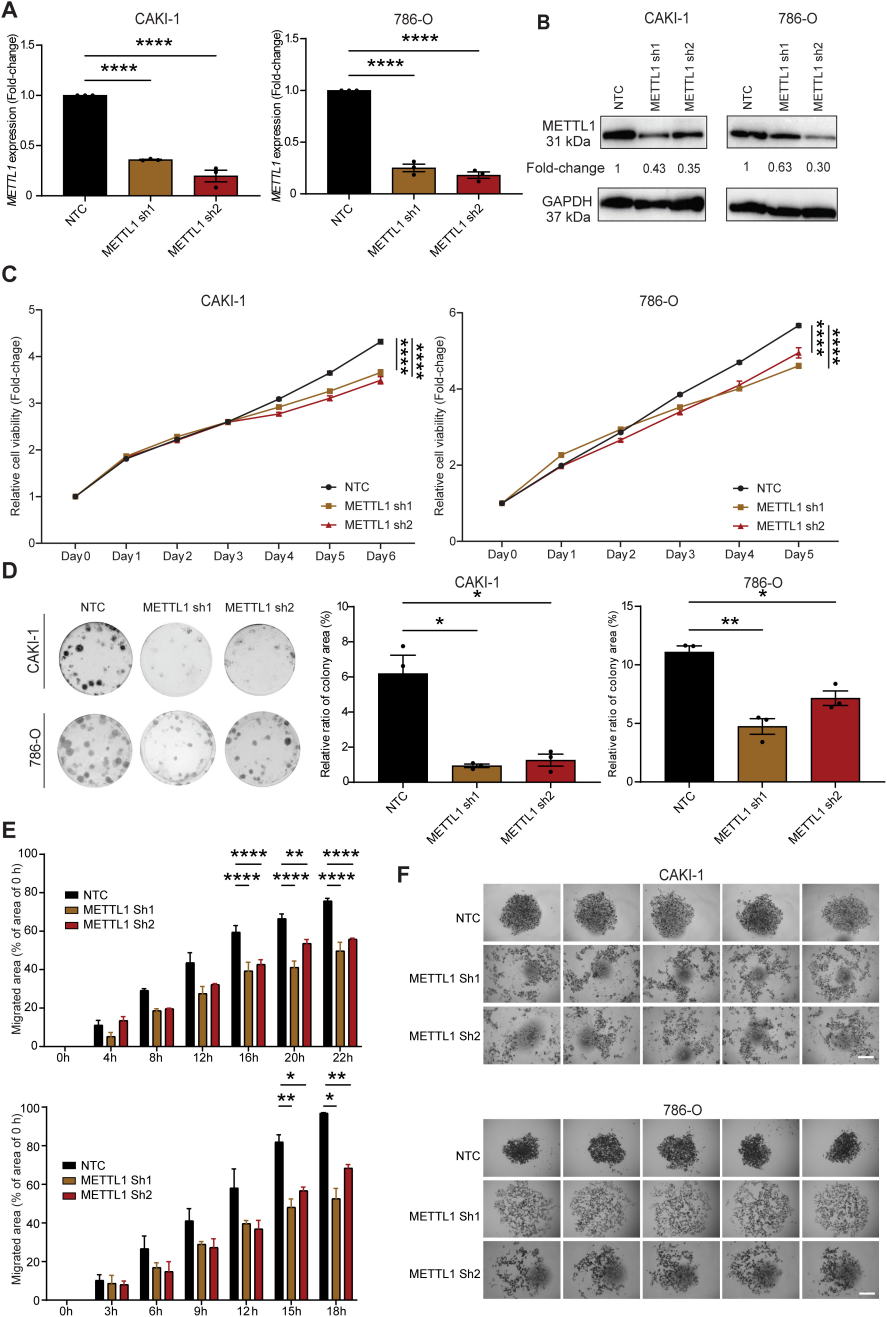
Knockdown of METTL1 suppresses proliferation, colony formation, and migration of KIRC cells. (A) Relative transcript levels of *METTL1* in CAKI‐1 and 786‐O cell lines following shRNA‐mediated depletion of METTL1 (sh1 and sh2) compared to a nontargeting shRNA control (NTC) measured by RT‐qPCR. (B) Representative western blotting of METTL1 protein levels in CAKI‐1 and 786‐O cells transduced with lentivirus expressing METTL1‐targeting shRNAs (sh1 and sh2) or NTC. Fold‐change of METTL1 expression normalized to the loading control (GAPDH) is shown relative to NTC. (C) Cell viability in METTL1‐depleted CAKI‐1 and 786‐O cells measured by CCK‐8 assay. Relative cell viability (fold‐change) are normalized to day 0. (D) Colony formation assay showing colony‐forming ability in METTL1‐depleted CAKI‐1 and 786‐O cells. The relative colony area ratio is shown for each group. (E) Wound healing assay showing migratory ability of METTL1‐depleted CAKI‐1 and 786‐O cells, with migrated area normalized to 0 h for each cell line. Data are presented as the mean **±** SEM from ≥3 biological replicates. For panels (A) and (D), significance was determined by one‐way ANOVA test. Two‐way ANOVA was performed to determine the significance in panels (C) and (E) by comparing METTL1 knockdown groups to the NTC control. Dunnett's Test was used for multiple comparisons of the means. *p < 0.05; **p < 0.01; ***p < 0.001; ****p < 0.0001. (F) Images of tumor mass that formed in the 3D culture spheroids following knockdown of METTL1 (sh1 and sh2) in CAKI‐1 and 786‐O cells compared to NTC. Scale bar, 200 μm.

To explore the functional role of METTL1 in KIRC, we depleted METTL1 using short hairpin RNAs (shRNA) in two KIRC cell lines, CAKI‐1 and 786‐O. Based on RT‐qPCR and western blotting assays, a significant decrease in transcript and protein levels of METTL1 (Figure 2A,B) led to significantly decreased cell proliferation and colony formation in CAKI‐1 and 786‐O compared to the control (Figure 2C,D). Wound‐healing assay revealed that *METTL1* knockdown significantly impaired cell migration (Figure 2E and supplementary material, Figure S2A). 3D culture demonstrated an impairment in tumor mass formation consequent to *METTL1* knockdown (Figure 2F). These findings suggest that METTL1 plays a role in KIRC maintenance by regulating cancer cell proliferation, clonogenicity, tumor mass formation, and migration.

**Figure 3:**
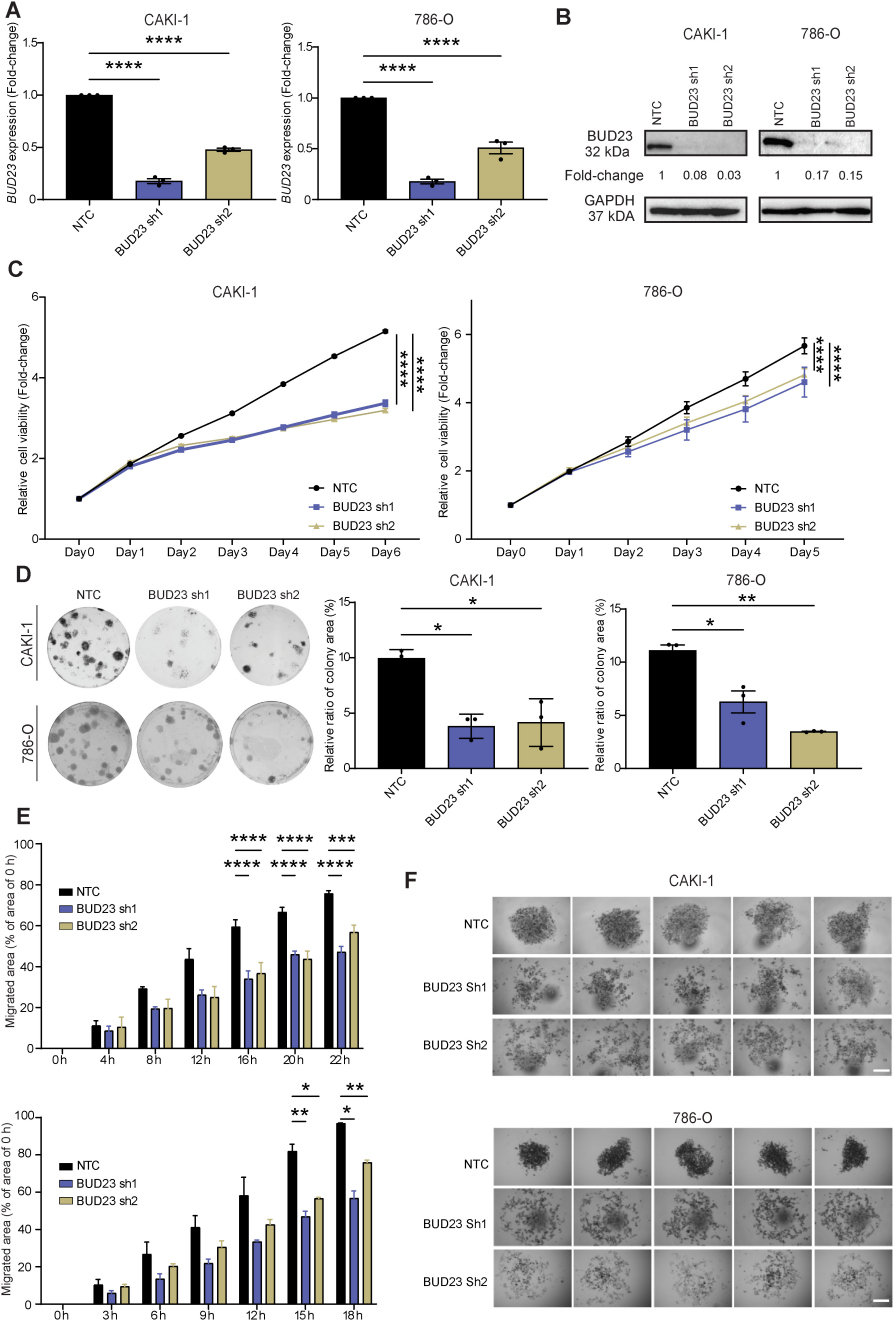
Knockdown of BUD23 suppresses proliferation, colony formation, and migration of KIRC cells. (A) Relative transcript levels of BUD23 in CAKI‐1 and 786‐O cell lines following shRNA‐mediated depletion of BUD23 (sh1 and sh2) compared to a nontargeting shRNA control (NTC) measured by RT‐qPCR. (B) Representative western blotting of BUD23 protein levels in CAKI‐1 and 786‐O cells transduced with lentivirus expressing BUD23‐targeting shRNAs (sh1 and sh2) or NTC. Fold‐change of BUD23 expression normalized to the loading control (GAPDH) is shown relative to NTC. (C) Cell viability in BUD23‐depleted CAKI‐1 and 786‐O cells measured by CCK‐8 assay. Relative cell viability (fold‐change) are normalized to day 0. (D) Colony formation assay showing colony‐forming ability in BUD23‐depleted CAKI‐1 and 786‐O cells. The relative colony area ratio is shown for each group. (E) Wound‐healing assay showing migratory ability of BUD23‐depleted CAKI‐1 and 786‐O cells, with migrated area normalized to 0 h for each cell line. Data are presented as the mean **±** SEM from ≥ 3 biological replicates. For panels (A) and (D), significance was determined by one‐way ANOVA test. Two‐way ANOVA was performed to determine significance for panels (C) and (E) by comparing BUD23 knockdown groups to the NTC control. Dunnett's Test was used for multiple comparisons of the means. *p < 0.05; **p < 0.01; ***p < 0.001; ****p < 0.0001. (F) Images of tumor mass that formed in the 3D culture spheroids following knockdown of BUD23 (sh1 and sh2) in CAKI‐1 and 786‐O cells compared to NTC. Scale bar, 200 μm.

We further investigated how BUD23 functions in KIRC. Similarly, shRNA‐mediated *BUD23* knockdown resulted in significantly reduced transcript and protein levels of BUD23 in CAKI‐1 and 786‐O cells (Figure 3A,B). Depletion of BUD23 significantly repressed the proliferation, clonogenicity, migration, and tumor mass formation in both cell lines (Figure 3C–F and supplementary material, Figure S2B). These results highlight that BUD23 is also important in the maintenance of KIRC tumors, indicating that upregulation of BUD23 confers oncogenic potential.

**Figure 4:**
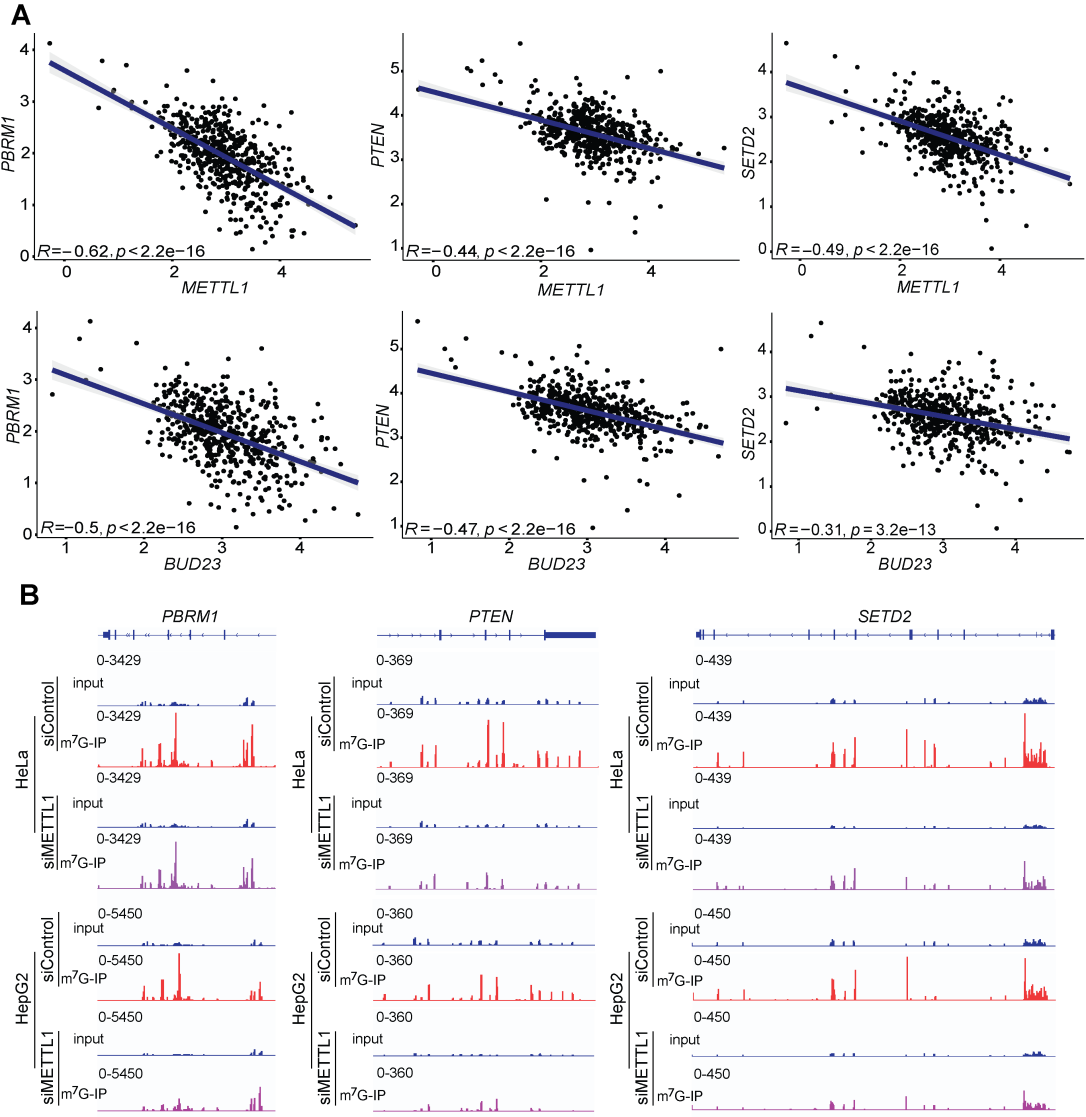
Negative correlation between *METTL1* and *BUD23* expression with *PBRM1*, *PTEN*, and *SETD2*, and observation of m^7^G methylation in these genes. (A). Correlation analysis of *METTL1* and *BUD23* expression with *PBRM1*, *PTEN*, and *SETD2* expression in KIRC. Each point represents log2‐FPKM gene expression values for individual patients in the TCGA dataset. The blue line indicates linear regression with Pearson's correlation coefficient (*R*) and *p* values shown. (B) Integrative genome viewer plots showing the m^7^G‐MeRIP‐seq peaks at *PBRM1, PTEN*, and *SETD2* in HeLa and HepG2 following stable knockdown of *METTL1* compared to control (GSE112276).

*METTL1* and *BUD23* overexpression are associated with dysregulation of MYC signaling and DNA repair pathways in KIRC (supplementary material, Figure S3). Using the TCGA KIRC datasets, we also demonstrated that *METTL1* and *BUD23* expression are negatively correlated with the expression of the most frequently dysregulated TSGs in KIRC, including polybromo 1 (*PBRM1*), phosphatase and tensin homolog (*PTEN*), and SET domain containing 2, histone lysine methyltransferase (*SETD2*) (Figure 4A) [16, 17, 18]. RT‐qPCR confirmed overall significantly higher expression of these TSGs in METTL1 knockdown, but not BUD23 knockdown cells relative to controls, consistent with METTL1's function as the mRNA m^7^G writer (supplementary material, Figure S4). We reanalyzed previously published m^7^G‐MeRIP‐seq datasets available for HeLa and HepG2 cells to determine the presence on m^7^G in *PBRM1*, *PTEN*, and *SETD2* transcripts [9]. We found that these transcripts are m^7^G‐methylated (Figure 4B and supplementary material, Figure S5) and m^7^G peaks decreased after *METTL1* knockdown in both cell lines compared to control (Figure 4B). These findings suggest that METTL1 may regulate the expression of TSGs (PBRM1, PTEN, and SETD2) in KIRC *via* an METTL1‐mediated internal m^7^G mRNA modification. IGF2BP3, an m^7^G reader, is known to bind m^7^G in mRNAs, promoting transcripts degradation [19]. Downregulation of these TSGs may be due to the action of IGF2BP3 in degrading m^7^G methylated mRNAs.

In summary, our study demonstrates the oncogenic roles of METTL1 and BUD23 in KIRC, as their expressions are linked to worse overall outcome in advanced KIRC and depletion of these m^7^G writers suppresses cell proliferation, colony formation, and migration *in vitro*. We show that *METTL1* and *BUD23* expression is negatively correlated with key TSGs (*PBRM1*, *PTEN*, and *SETD2*) in KIRC. Furthermore, m^7^G‐MeRIP‐seq analysis indicates the METTL1‐mediated m^7^G modification affects mRNAs encoded by these TSGs, underscoring the involvement of m^7^G‐dependent pathways in KIRC pathogenesis. These findings clearly indicate the potential of METTL1 and BUD23 upregulation as prognostic biomarkers, and suggest potential mechanism of how these m7G writers contribute to KIRC maintenance *via* an m^7^G‐dependent manner. Our study establishes a foundation for future *in vivo* and mechanistic studies to further elucidate the roles of METTL1 and BUD23 in KIRC and assess their potential as therapeutic targets.

## Author contributions statement

JJ‐LW conceived the idea. AS and JT performed experiment and analysis. AS performed bioinformatic analysis. XSX and RPT performed scratch assay. YF designed, performed the 3D culture and analyzed results. AS, YF and JJ‐LW wrote the article.

## Supporting information


Supplementary materials and methods
**Figure S1.** Multivariate analysis of *METTL1* and key clinicopathological features in KIRC
**Figure S2.** Representative images from wound healing assays showing migratory ability of (A) METTL1 and (B) BUD23‐depleted CAKI‐1 and 786‐O cells, with migrated area normalized to 0 h for each cell line
**Figure S3.** Correlation between *METTL1* (A) and *BUD23* (B) expression and cancer hallmark pathways
**Figure S4.** Relative expression of *PBRM1, PTEN*, and *SETD2* in CAKI‐1 and 786‐O cells subjected to *METTL1* and *BUD23* knockdown (METTL1 sh1, METTL1 sh2, BUD23 sh1 and BUD23 sh2) compared to non‐targeting shRNA control (NTC)
**Figure S5.** Integrative genome viewer plots showing the m^7^G ‐MeRIP‐seq peaks at *PBRM1, PTEN*, and *SETD2* in HeLa and HepG2 cells (GSE112276)
**Table S1.** Sequences of shRNAs against non‐targeting control, *METTL1* and *BUD23*
**Table S2.** RT‐qPCR Primers used in this study
**Table S3.** Antibodies used in this study

## Data Availability

The results shown here are in part based on data generated by the TCGA Research Network (https://cancergenome.nih.gov/) and GSE112276 (https://www.ncbi.nlm.nih.gov/geo/query/acc.cgi?acc=GSE112276). The processed data that support the findings of this study are available from the corresponding author upon reasonable request.
