# Supplementary material for: Overexpression of m7G writers METTL1 and BUD23 confers oncogenicity in kidney renal clear cell carcinoma
Source: J Pathol. 2025 Jul 18;267(1):1–9. doi: 10.1002/path.6453 (PMC12337808; doi:10.1002/path.6453)
Supplement: Supplementary file 1 — Supplementary materials and methods Figure S1. Multivariate analysis of METTL1 and key clinicopathological features in KIRC Figure S2. Representative images from wound healing assays showing migratory ability of (A) METTL1 and (B) BUD23‐depleted CAKI‐1 and 786‐O cells, with migrated area normalized to 0 h for each cell line Figure S3. Correlation between METTL1 (A) and BUD23 (B) expression and cancer hallmark pathways Figure S4. Relative expression of PBRM1, PTEN, and SETD2 in CAKI‐1 and 786‐O cells subjected to METTL1 and BUD23 knockdown (METTL1 sh1, METTL1 sh2, BUD23 sh1 and BUD23 sh2) compared to non‐targeting shRNA control (NTC) Figure S5. Integrative genome viewer plots showing the m7G ‐MeRIP‐seq peaks at PBRM1, PTEN, and SETD2 in HeLa and HepG2 cells (GSE112276) Table S1. Sequences of shRNAs against non‐targeting control, METTL1 and BUD23 Table S2. RT‐qPCR Primers used in this study Table S3. Antibodies used in this study [file PATH-267-1-s001.docx]

**Overexpression of m^7^G writers METTL1 and BUD23 confers oncogenicity in kidney renal clear cell carcinoma**

A Su *et al.* *J Pathol* <https://doi.org/10.1002/path.6453>

**Supplementary materials and methods**

**Supplementary Figures S1–S5**

**Supplementary Tables S1–S3**

Reference numbers refer to the main text list.

**Supplementary materials and methods**

**m^7^G-MeRIP-seq analysis**

Data quality was checked using FastQC [20]. Adapter sequences and low-quality reads were trimmed by Trimmomatic [21]. Clean single-end reads were then mapped to the human reference genome (hg38 from Ensembl Release-113) using the STAR aligner [22]. Uniquely mapped reads were filtered with SAMtools for downstream analysis [23]. MACS2 was used to call m^7^G summit peaks by comparing m^7^G immunoprecipitation RNA (m^7^G-IP) with input control RNA [24]. Significant peaks with a false discovery rate < 0.05 and enrichment score > 5 were selected. Candidate genes were visualized using the Integrative Genomics Viewer [25].

**Short hairpin RNA (shRNA) lentiviral vector cloning and production**

To cloned shRNAs (supplementary material, Table S1) into the pLKO.1-puro vector (Addgene, Watertown, MA, USA), the 100 ng digested pLKO.1-puro vectors and 4 ng annealed shRNA oligonucleotides were ligated with 1 μl T4 Rapid Ligase (Promega, Madison, WI, USA) and 5 μl 2× Rapid Ligase buffer (Promega), and 1 μl 40 mM ATP (New England Biolabs, Ipswich, MA, USA) a 10-μl reaction at room temperature for 45 min. Competent DH5α cells were thawed on ice for 30 min. Then, 5 μl of ligation mixture was added to 50 μl DH5α cells and incubated on ice for 40 min, followed by heating at 42 °C for 30 sec, then placing back on ice for 2 min. Super optimal broth with catabolite repression (S.O.C.) medium (Invitrogen, Waltham, MA, USA) was added (1:10 ratio), and the cells were shaken at 37 °C, 200 RPM for 1 h. Afterwards, cells were plated on LB agar plates containing 100 μg/ml ampicillin (Astral Scientific, Tarren Point, NSW, Australia) and incubated at 5% CO_2_ and 37 °C overnight. Following incubation, single transformed DH5α cell colonies were picked and cultured in LB medium containing 100 μg/ml ampicillin (Astral Scientific) at 37 °C, 200 rpm overnight. Plasmid DNA were extracted using a Zyppy™ Plasmid Miniprep Kit (Zymo Research, Irvine. CA, USA).

HEK-293T cells were transfected by calcium phosphate using the appropriate expression vector. Cells were cultured in DMEM (ThermoFisher, Waltham, MA, USA) supplemented with 10% FBS (Bovogen, East Keilor, Victoria, Australia), and 1% penicillin/streptomycin (ThermoFisher) and incubated overnight at 5% CO_2_ and 37 °C. At 1 h before transfection, the medium was replaced with fresh medium containing 25 µM chloroquine (Sigma-Aldrich, St. Louis, MO, USA). For transfection, a mixture containing 6.5 μg pMDL-g/prre vector, 2.5 μg pRSV-Rev vector, 3.5 μg pMD2.VSV-G vector, 15 μg shRNA-inserted pLKO.1.ezap vector, 2.5 M CaCl_2_, and 1/10 TE buffer was added dropwise to a tube containing 300 mM phosphate buffer and 2× HEPES buffer (ThermoFisher) placed on a vortex at maximum speed. The resulting solution was then added dropwise to the cells and incubated overnight. The medium was replaced with fresh medium containing 2 mM sodium butyrate (Sigma-Aldrich) and incubated for another 24 h. Cell supernatant was centrifuged at 300× *g* for 5 min. The lentivirus-containing supernatant was filtered using a 0.45 μm filter and stored at −80 °C.

**Lentivirus transduction**

Cells were transduced by lentivirus via spinoculation. 2.5 × 10^5^ cells in 500 µl of culture medium with 8 µg/ml polybrene (Sigma-Aldrich) were placed into 5-ml polystyrene capped tubes. Then, 500 µl of lentivirus was added to the tube. The tubes were spinoculated at 350 × *g* at 22 °C for 90 min. After spinoculation, the tubes were vortexed briefly and incubated at 5% CO_2_ and 37 °C for 4 h. Cells were then centrifuged at 350 × *g* for 5 min. The viral supernatant was discarded. Cells were resuspended in fresh medium and incubated at 5% CO_2_ and 37 °C for 48 h. After 48 h, transduced cells were selected by puromycin (ThermoFisher).

***In vitro* functional assay**

CCK8 assay (Abcam, Cambridge, UK) was performed following the manufacturer’s protocol to determine cell proliferation. 1,000 cells were seeded in 96-well plates in quintuplicate at 37 °C and 5% CO_2_. After 24 h, 5 µl of WST8 was added per well and absorbance measured at 530 nm after 2 h on day 0. Measurements were repeated for 4–6 days.

Colony formation assay was performed by seeding 100 transduced 786-O cells and 300 transduced CAKI-1 cells per well in a 6-well plate in triplicate at 5% CO_2_ and 37 °C for 10−14 days. Colonies were fixed, stained with Giemsa (Sigma Aldrich), and analyzed using ImageJ [26].

The wound-healing assay was performed to assess cell migration. 786-O cells (2,500 cells per well) and CAKI-1 cells (9,000 cells per well) were seeded in triplicate in 96-well plates. When cells reached 90–100% confluence, a scratch was made using the Incucyte® Woundmaker Tool (Sartorius, Göttingen, Germany). Plates were placed within Incucyte® SX5 Zoom microscope (Sartorius) inside the incubator and images were captured at 0 to 24 h. Wound closure was calculated as the migrated area of the cells, relative to the measured scratch area at day 0, quantified using ImageJ.

**3D cell culture**

CAKI-1 and 786O cells were seeded at 5,000 cells per 30-μl droplet on the lid of a 10-cm tissue culture dish to form hanging drops. The lid was then inverted onto the PBS-filled base of the dish and incubated at 37 °C in a humidified incubator until 3D spheroids were formed. Images were captured using a ZEISS Axiovert inverted microscope (Oberkochen, Germany).

**Correlation between m^7^G writer gene expression and cancer hallmark pathway**

Cancer hallmark gene sets were obtained from MSigDB (https://www.gsea-msigdb.org/gsea/msigdb/). Gene Set Variation Analysis (GSVA) was performed as previously reported [14]. The Pearson Correlation Coefficient (PCC) between the expression of m^7^G writers and pathway activity was calculated using the “Hmisc” R-package (<https://cran.r-project.org/web/packages/Hmisc/index.html>).


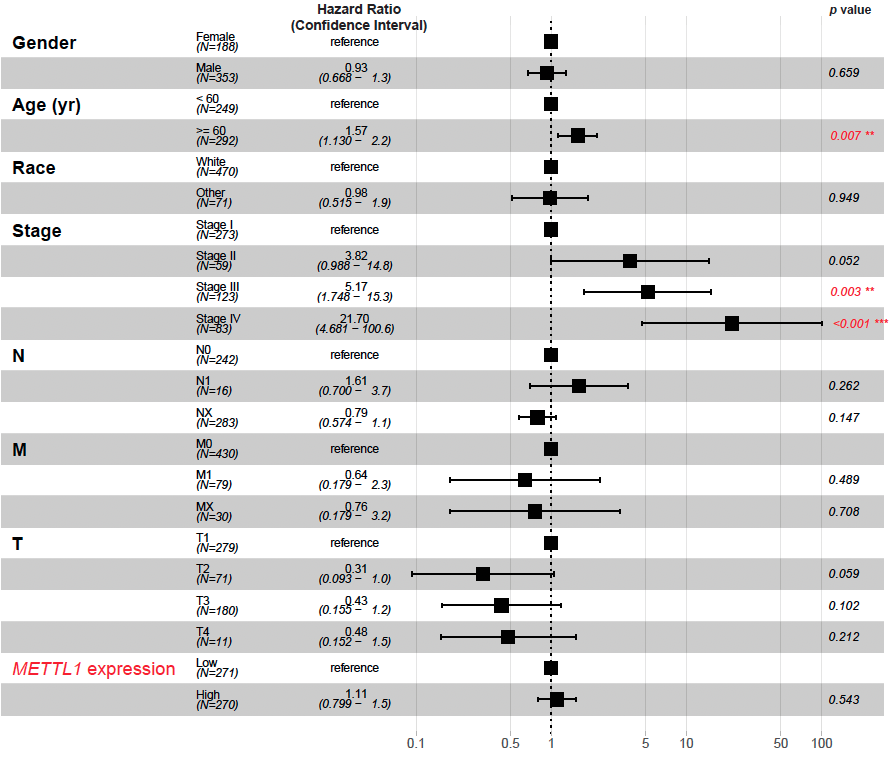


**Figure S1.** **Multivariate analysis of *METTL1* and key clinicopathological features in KIRC.** Significance is denoted by *p <*0.05 with a log-rank test.


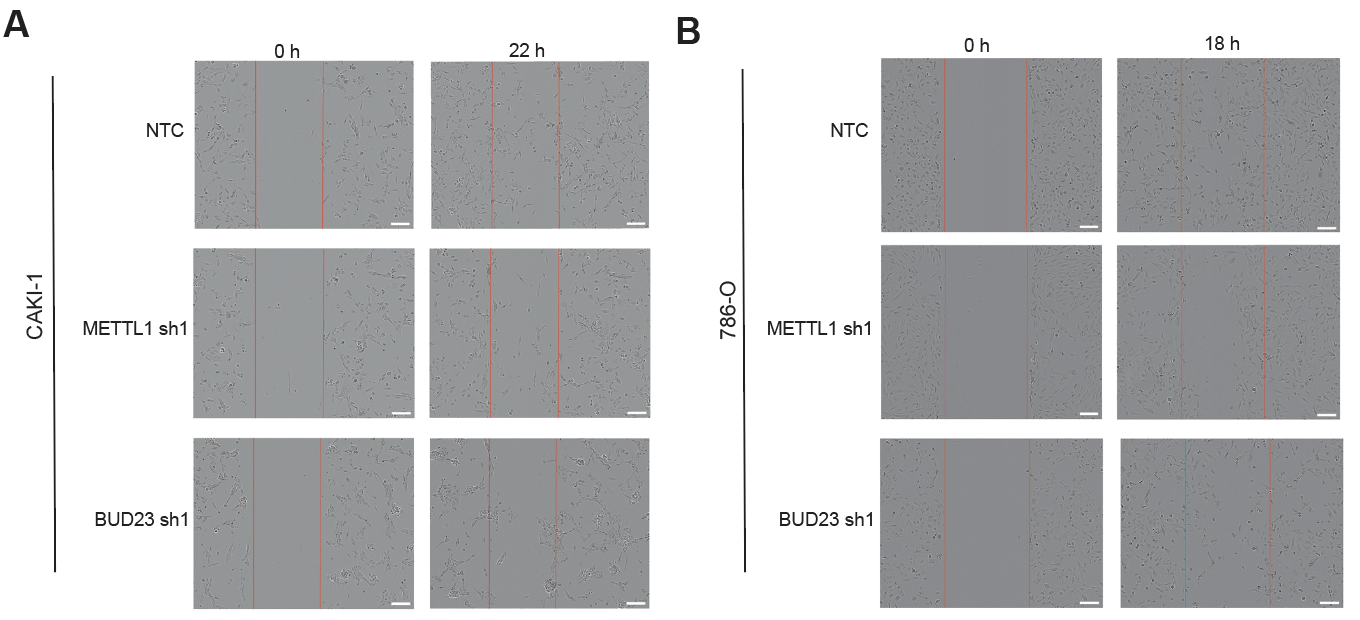


**Figure S2. Representative images from wound-healing assays showing migratory ability** **of METTL1 and BUD23-depleted (A) CAKI-1 and (B) 786-O cells, with migrated area normalized to 0 h for each cell line.** Scale bars, 150 μm.


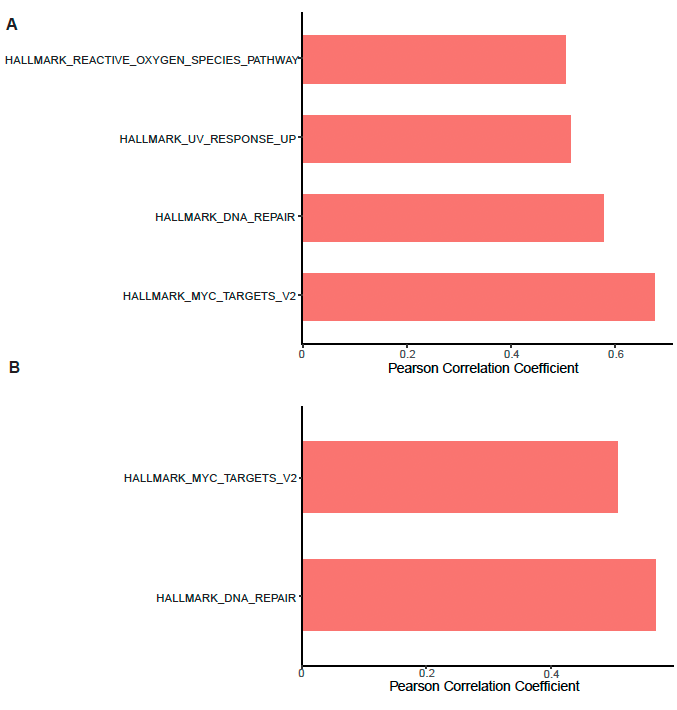


**Figure S3. Correlation between (A) *METTL1* and (B) *BUD23* expression and cancer hallmark pathways.**


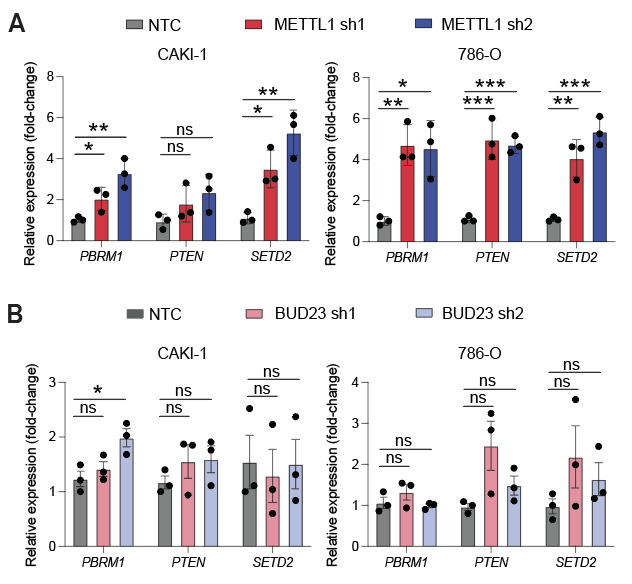


**Figure S4.** **Relative expression of *PBRM1, PTEN,* and *SETD2* in CAKI-1 and 786-O cells subjected to *METTL1* and *BUD23* knockdown (METTL1 sh1, METTL1 sh2, BUD23 sh1 and BUD23 sh2) compared to nontargeting shRNA control (NTC).** Fold-change was calculated based on the relative expression of each gene to (A) METTL1 and (B) BUD23, respectively, normalized to loading control. Data are presented as the mean ± SEM from  ≥ 3 biological replicates. Significance was determined by one-way ANOVA and Dunnett’s Test was used for multiple comparisons of the means. **p* < 0.05; ***p* < 0.01; ****p* < 0.001; ns, not significant.


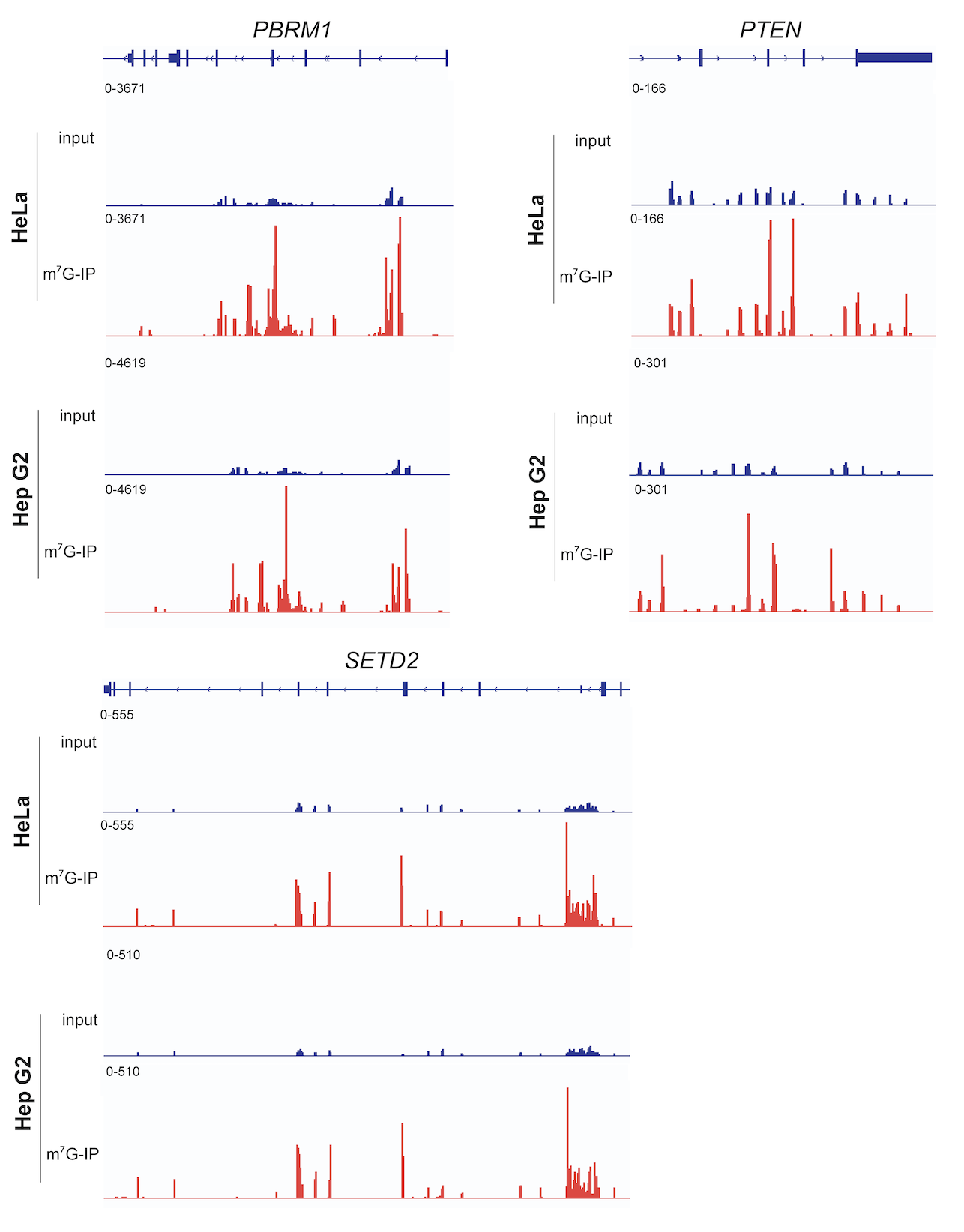


**Figure S5. Integrative genome viewer plots showing the m^7^G -MeRIP-seq peaks at *PBRM1, PTEN,* and *SETD2* in HeLa and HepG2 cells (GSE112276).**

**Table S1.** Sequences of shRNAs against nontargeting control, *METTL1,* and *BUD23*.

| Target | Sequence |
| --- | --- |
| Ath-miR-159  (nontargeting control) | **Forward:**  CCGGTTTGGATTGAAGGGAGCTCTTCAAGAGAGAGCTCCCTTCAATCCAAACTTTTTTC  **Reverse:**  AATTGAAAAAAGTTTGGATTGAAGGGAGCTCTCTCTTGAAGAGCTCCCTTCAATCCAAA |
| METTL1-sh1 | **Forward:**  CCGGGATGACCCAAAGGATAAGAAACTCGAGTTTCTTATCCTTTGGGTCATCTTTTG  **Reverse:**  AATTCAAAAAGATGACCCAAAGGATAAGAAACTCGAGTTTCTTATCCTTTGGGTCATC |
| METTL1-sh2 | **Forward:**  CCGGCCCACATTTCAAGCGGACAAACTCGAGTTTGTCCGCTTGAAATGTGGGTTTTTG  **Reverse:**  AATTCAAAAACCCACATTTCAAGCGGACAAACTCGAGTTTGTCCGCTTGAAATGTGGG |
| BUD23-sh1 | **Forward:**  CCGGGCCCTGTTACCTGCTGGATCTCGAGATCCAGCAGGTAACAGGGCTTTTTG  **Reverse:**  AATTCAAAAAGCCCTGTTACCTGCTGGATCTCGAGATCCAGCAGGTAACAGGGC |
| BUD23-sh2 | **Forward:**  CCGGGTCAGATGAAGGGCACTATCTCGAGATAGTGCCCTTCATCTGACTTTTTG  **Reverse:**  AATTCAAAAAGTCAGATGAAGGGCACTATCTCGAGATAGTGCCCTTCATCTGAC |

**Table S2.** RT-qPCR primers used in this study.

| **Primer** | **Sequence** |
| --- | --- |
| *METTL1* | **Forward:**  TTCTTCTACAAGGGCCAGCT  **Reverse:**  ATTCGCCACTTGTGCTTTGT |
| *BUD23* | **Forward:**  ATACGTTCGCAACTCACGGA  **Reverse:**  CCAGCAGGTAACAGGGCTTA |
| *PBRM1* | **Forward:**  TGTCAGCGGGGACTTTGATG  **Reverse:**  AGCTCACAGAGAAGTCTGCC |
| *PTEN* | **Forward:**  AGCATTTGCAGTATAGAGCGTGC  **Reverse:**  AGCCTCTGGATTTGACGGCT |
| *SETD2* | **Forward:**  AGCGAATGCAGTGTGAGTGT  **Reverse:**  TCCCCATTTGGACACCGAGA |
| *GAPDH* | **Forward:**  TCAAGGCTGAGAACGGGAAG  **Reverse:**  GGACTCCACGACGTACTCAG |

**Table S3.** Antibodies used in this study.

| Specificity | Species | Clonality | Cat No. | Company | Dilution |
| --- | --- | --- | --- | --- | --- |
| METTL1 | Rabbit | Poly | ab157097 | Abcam | 1:1,000 |
| BUD23 | Rabbit | Poly | ab97911 | Abcam | 1:500 |
| GAPDH | Mouse | Mono | ab9485 | Abcam | 1:5,000 |
| Rabbit IgG | Donkey | Poly | AP192P | Merck Millipore | 1:5,000 |
| Mouse IgG | Donkey | Poly | AP182P | Merck Millipore | 1:5,000 |
